# The Association between Neighborhood Context, Allostatic Load, and Metabolic Dysfunction-Associated Steatosis Liver Disease in Mexican-origin Farmworkers along the Southern Arizona US/Mexico Border

**DOI:** 10.21203/rs.3.rs-6448142/v1

**Published:** 2025-05-13

**Authors:** Adriana Maldonado, Emma Torres, Melissa Flores, Mariella Rodriguez, Edgar A. Villavicencio, Rogelio Torres, Idolina Castro, Felicitas Torres, Julio C. Loya, Naim Alkhouri, Scott Carvajal, David O. Garcia

**Affiliations:** University of Arizona; Campesinos sin Fronteras; University of Arizona; University of Arizona; University of Arizona; Campesinos sin Fronteras; Campesinos sin Fronteras; Campesinos sin Fronteras; University of Arizona; Arizona Liver Health Chandler; University of Arizona; University of Arizona

**Keywords:** Neighborhood context, MASLD, Allostatic load, Farmworkers, Mexicans

## Abstract

**Background:**

Mexican-origin farmworkers are at an increased risk of Metabolic dysfunction-associated steatosis liver disease (MASLD). They are also susceptible to living in neighborhoods with higher levels of unfavorable social, physical, and economic conditions. The effects on health due to these neighborhood conditions are suggested to be reflected through Allostatic Load (ALoad), the wear and tear of the body due to chronic stress. This study examined associations between perceived neighborhood environment, ALoad, and MASLD.

**Methods:**

Data were collected from a community-based sample of 151 Mexican-origin farmworkers residing in the Southern Arizona U.S./Mexico border region. Self-reported data on six dimensions of neighborhood context was collected. Allostatic load was calculated as an index of physiological dysregulation. Hepatic steatosis and fibrosis were assessed by liver stiffness measurements (LSM) and controlled attenuation parameter (CAP) scores via FibroScan^®^. MASLD were identified as having a CAP score of ≥288 dB/m.

**Results:**

The mean age was 49.7 ±14.1 years, mean BMI 31.9 ±6.5 kg/m2, and 9.9% had type 2 diabetes. The mean CAP score was, *M*=265.8 ±61.0 with 41.1% of the sample exhibiting MASLD status. Perceived neighborhood violence was not associated with MASLD status; however, it was negatively associated with ALoad, (*p*=0.003). ALoad was a negative mediator between perceived violence and MASLD status (*p*=0.004).

**Conclusion:**

The results of this study may inform policy and programmatic efforts, such as context for the development of culturally relevant strategies to reduce Mexican-origin farmworkers’ risk for MASLD that are highly responsive to the structural and systemic forces that shape their lived experience.

## BACKGROUND

Metabolic dysfunction-associated steatosis liver disease (MASLD; formerly known as non-alcoholic fatty liver disease), is the leading cause of liver-related morbidity and mortality worldwide.(1) MASLD, characterized by the buildup of excess fat in the liver tissue, is not caused by alcohol consumption and may progress into metabolic dysfunction-associated steatohepatitis (MASH) and ultimately result in cirrhosis and/or hepatocellular carcinoma.(2) In the Southern Arizona US/Mexico border region, estimates indicate that the overall prevalence of MASLD among Mexican-origin adults with overweight and obesity is nearly 50%;(3) an estimate that is well above the 43% prevalence reported at a national level.

Despite the disproportionate burden of MASLD experienced by Mexican-origin adults in general, studies exploring MASLD risk among Mexican-origin farmworkers do not exist. This is surprising not only because most of the hired farmworkers in the U.S. are of Mexican descent,(4) but because they are also at an increased risk of experiencing many MASLD related risk factors. Specifically, results from the scant literature exploring chronic disease risk among farmworkers suggest that compared to national estimates, the prevalence of hypertension (HTN), type 2 diabetes (T2D), hypercholesterolemia, and obesity are significantly higher among farmworkers.(5–9) In Yuma County, Arizona’s largest agricultural producing county in the state, rates of diagnosed MASLD risk factors among farmworkers are high with 21% for HTN and 16% for T2D.(10) This is of particular concern in the Southern Arizona region, as liver disease and cancer (including liver cancer) are among the leading causes of death in Mexican-origin adults.(11)

Despite compelling evidence that the social and built environment affect population health as much as individual-level characteristics, a neighborhood’s social and built environment factors have rarely been considered in cancer-related research.(12) Mexican-origin Hispanics tend to reside in neighborhoods with higher levels of social, physical, and economic inequities that increases their risk for obesity and T2D, known risk factors for MASLD.(13) A neighborhood’s social and built environment can impact an individual’s health via different pathways including: (1) availability of physical resources, (2) access to healthy and affordable food, (3) availability of adequate and preventive care, and (4) exposure to violence and stressful life events.(14, 15) In fact, it has been previously suggested that a neighborhood’s effects on health might operate through allostatic load (ALoad), which is the wear and tear on the body due to stress.(16, 17) ALoad, the chronic activation of the stress response in the presence of chronic stressors, may lead to physiologic dysregulation and increases the risk for obesity, T2D, heart disease, and cancer. (18) While studies have found an association between ALoad and external socio-environmental stressors,(19, 20) measures of ALoad have rarely been included in farmworker’s health studies.(21, 22) Thus, building upon existent research and addressing important gaps, the purpose of this study is to examine associations between neighborhood context, ALoad, and MASLD in a cross-sectional sample of Mexican-origin farmworkers.

## METHODS

### Study Setting

Study activities took place in Yuma County, AZ – “the lettuce capital of the world”. Yuma County, located along the U.S.-Mexico border, is a prime destination for farmworkers due to climatic conditions that allow for various winter agricultural opportunities, including lettuce, broccoli, cauliflower, citrus, and melons.(10) Yuma County has the largest farmworker population in the state of Arizona with estimates indicating that 6,470 individuals were employed in 2023.(23) The annual mean wage for farmworkers in 2023 Yuma was $34,420, which is just above the poverty guidelines for a household of four ($30,000). (24) While no comprehensive report exists describing farmworker’s characteristics in Yuma County, results from a community-based survey (n = 298) indicate that majority of farmworkers are foreign-born (96% from Mexico), are married (80%), are legal permanent residents (77%), and have an education level of less than 6^th^ grade (72%).(10)

### Study Design, Sample, and Recruitment

A cross-sectional study was conducted to assess the relationship between neighborhood context, ALoad, and MASLD in a sample of 151 farmworkers from Yuma County, AZ. Eligible participants 1) self-identified as Mexican-origin farmworkers, 2) were 18 years of age or older, 3) had resided or worked in Yuma County for at least 1 year, 4) were able to provide informed consent, and 5) had the ability to speak, read, and write in Spanish/English. Farmworkers were not eligible to participate if they reported: 1) ongoing or recent alcohol consumption (≥21 standard drinks on average per week for men and ≥14 standard drinks on average per week for women); 2) had a history of exposure to hepatotoxic drugs; or 3) were previously diagnosed with liver disease or liver cancer.

Participant recruitment was primarily led by Campesinos Sin Fronteras (CSF), a non-profit organization dedicated to educating, serving and advocating for Yuma County residents, in particularly farmworker families, to prevent chronic disease, injury, illness and promote overall well-being. CSF helped the university-based research team to identify natural groups in which potential participants could be identified and recruited. Three strategies were used to recruit participants including (1) face-to-face interactions between community health workers and potential participants, (2) social media posts, and (3) word-of-mouth.

### Study Procedures

Data collection occurred from May to August 2023. Study visits took place at CSF’s offices in Gadsden, AZ. Prior to data collection, informed consent was obtained. Eligible participants completed a 90 minute in-person visit in which MASLD status, anthropometric measures, allostatic load biomarkers, and self-reported questionnaires were obtained. Study visits and self-reported questionnaires were all completed in Spanish which was the participants’ language of choice. After completing all data collection procedures, participants were compensated $50 for their time with an additional $10 to offset any additional costs associated with travel. The University of Arizona Institutional Review Board (IRB # STUDY00002483) approved all study materials and research protocol.

### Measures

#### Metabolic Dysfunction-Associated Steatosis Liver Disease

MASLD status was identified via vibration-controlled transient elastography (FibroScan^®^ 430 MINI+). The FibroScan^®^ derives the median for continuous attenuation parameter (CAP) and liver stiffness measurement (LSM) values with higher values indicating higher amount of liver fat and fibrosis.(25) Participants identified with MASLD had CAP scores of ≥288 dB/m.(26) FibroScan^®^ examinations were considered valid if participants fasted for at least 3 hours, more than 10 individual LSM measures were collected, and the Interquartile Range (IQR)/median on LSM values were less than 30%.(25)

#### Neighborhood Context

Neighborhood context was assessed using the Neighborhood Scale.(27) The scale evaluates seven neighborhood dimensions including: aesthetic quality, walking environment, availability of healthy foods, safety, violence, social cohesion, and activities with neighbors. A total score was calculated for each subscale by averaging all items within the scale. A subscale score was only given to participants with complete data. Original responses for each item ranged from 1 to 4 (1 = often, 2 = sometimes, 3 = rarely, and 4 = never). All items for the scale except for items one and two in the aesthetic quality subscale were reverse coded for analysis. Larger means for the aesthetic quality, walking environment, availability of healthy foods, safety, social cohesion, and activities with neighbors subscales meant greater neighborhood aesthetic quality, walkability, availability of healthy foods, safety, social cohesion, and activities with neighbors. While for the violence subscale, larger means related to worse neighborhood violence.

#### Allostatic Load

ALoad, the cumulative multisystem physiological dysregulation resulting from chronic exposure to stress, was derived from ten biomarkers of cardiometabolic risk, glucose metabolism, cardiopulmonary functioning, parasympathetic functioning, and inflammation.(28–31) These biomarkers included systolic and diastolic blood pressure, body mass index (BMI), glycohemoglobin, total cholesterol, high-density lipoprotein (HDL) cholesterol, total/HDL cholesterol ratio, C-reactive protein, albumin, and creatinine clearance.(29) Each biomarker was categorized as high-, moderate-, or low-risk following clinically relevant cut-points (**Table 1**). An ALoad index was then calculated by assigning one point for the high-risk category, a half point for moderate-risk, and zero points for low-risk. A half point was added to the ALoad index if participants reported taking medication for hypertension, diabetes, and/or cholesterol and who had a low-risk value for blood pressure, glycohemoglobin, or lipids.(32)

#### Anthropometric Measures

Blood pressure, height, and weight were collected using standardized methods.(33) A digital blood pressure monitor (Omron HEM-907XL) was used to measure systolic and diastolic blood pressure. Two consecutive blood pressure measurements were obtained by trained research staff. If the measurements for systolic blood pressure differ by > 10 mmHg or diastolic blood pressure differed by > 6 mmHg, a third measurement was obtained. The average of the two measurements meeting the criteria for systolic and diastolic blood pressure were used for analyses purposes. Using a wall-mounted stadiometer (ShorrBoard) participants’ height was measured without shoes, twice to the nearest 0.1 cm. In cases where the height measurements differed by more than 0.5 cm, a third measurement was taken. Without shoes and in participants’ street clothes, participants’ weight was measured twice on a calibrated digital scale (Seca 8760) to the nearest 0.1 kg. A third measurement was taken if the two measurements diverged by more than 0.2 kg. The average of the two measurements meeting the criteria for height and weight were used to calculate body mass index (BMI). BMI was computed by dividing the body weight in kilograms by squared height in meters (kg/m^2^).

#### Demographic Characteristics and Acculturative Stress

Descriptive variables including age, sex, nativity, marital status, employment status, annual household income, educational attainment, health insurance status, and primary language spoken at home were collected. In addition, acculturative stress was measured using the Societal, Attitudinal, Environmental, and Familial Acculturative Stress Scale (SAFE).(34)

### Statistical Analyses

Multivariable logistic and linear regressions were used to assess the associations between neighborhood variables and the outcomes: ALoad, MASLD status (0 = no disease; 1 = MASLD), and Fibrosis. Fibrosis was log-transformed to normalize model residuals. Both adjusted and unadjusted models were estimated for all paths. Final models were adjusted for age, sex, education, nativity, and acculturative stress.(34)

Mediation of perceived neighborhood violence on MASLD and Fibrosis by ALoad was estimated using a two-equation solution where we assessed whether the ab path was not equal to zero for M=aX+eM, and Y=c'X+bM+ey where M is ALoad, X is a neighborhood variable (e.g. perceived neighborhood violence) and *Y* is the log odds of MASLD status or Fibrosis. Bias corrected and accelerated confidence intervals were calculated with n = 10,000 bootstraps. Mediation analyses were estimated with fully adjusted models with the exception of education due to small cell sizes in several categories that restricted accurate standard error estimation. Results did not change in substantive estimates or interpretation when removing this covariate.

## RESULTS

### Sample Characteristics

The sample (N = 151) had a mean age of 49.7 years (SD = 14.1) and was majority female (n = 90, 59.6%). Nearly all participants were born outside of the U.S. (90.7%; n = 137). MASLD was present in 41.1% of participants (n = 62). The sample had an average BMI of 31.9 kg/m^2^ (SD = 6.5), weight of 85.4 kg (SD = 20.4), and waist circumference of 105.4 cm (SD = 15.6). Regarding comorbid conditions, 28.5% (n = 43) of participants self-reported having HTN and 9.9% (n = 15) reported having T2D. Most participants were married (66.9%; n = 101) and unemployed (82.1%; n = 124), with a majority reporting an annual household income of less than $29,999 a year (84.8%; n = 128). Most participants reported having completed less than ninth grade (65.6%; n = 99), however most participants reported having active health insurance (58.9%; n = 89). BMI, weight, waist circumference, and ALoad were higher in those with MASLD versus not (all *p*'s < 0.001; **Table 2**). Rates of liver fibrosis were greater in the group with MASLD (*p* = 0.006; **Table 2**). See Table 2 for other relevant demographic characteristics of our sample. The prevalence of steatosis was 13%, 5.3%, and 45% for steatosis stages S1, S2, and S3 respectively. Almost 9% of participants had significant fibrosis based on kPa values (**Table 3**).

### Main Analyses

In both unadjusted and adjusted analyses, neighborhood variables were not associated with MASLD status or Fibrosis. See **Table 4** for relevant model estimates.

Perceived neighborhood violence was negatively associated with ALoad in both unadjusted, *b* = −0.15, *se* = 0.05, *p* = 0.001; 95% confidence interval (CI) (−0.24, −0.06), and adjusted models *b* = −0.14, *se* = 0.05, *p* = 0.003; 95% CI (−0.24, −0.05). No other neighborhood variables were associated with ALoad in either unadjusted or adjusted analyses. Mediation analyses revealed that ALoad was a non-zero mediator between perceived neighborhood violence and MASLD status, average mediated effect = −0.02, 95% CI (−0.04, −0.01), *p* = 0.004; total effect = −0.04, 95% CI (−0.06, 0.01), *p* = 0.06). No other non-zero mediation estimates were found. See **Table 5**.

## DISCUSSION

Using data from a cross-sectional sample of Mexican-origin farmworkers, this study aimed to examine relationships among neighborhood context, ALoad, and MASLD. While 41% of the sample was identified as having MASLD, neighborhood context was not associated with MASLD status. However, perceived neighborhood violence was negatively associated with ALoad. When mediation effects were explored, ALoad was found to be a non-zero mediator between perceived neighborhood violence and MASLD status. To the best of our knowledge, this is the first study to examine the association between neighborhood context, ALoad and MASLD in Mexican-origin farmworkers. Thus, additional efforts are warranted to identify the mechanistic pathways through which neighborhood context influenced Mexican-origin farmworkers risk for MASLD. Nevertheless, study findings suggest the current need for multilevel context-specific interventions to consider environmental factors to promote liver disease prevention and treatment efforts among Mexican-origin farmworkers.

Though previous work suggests that characteristics of the neighborhood environment are associated with adverse cardio-metabolic outcomes,(35–37) in the current study, perceived neighborhood environment was not directly associated with MASLD status nor Fibrosis levels. The results of this study are congruent with some previous studies where no significant association between neighborhood environment and levels of HbA1C(38) or metabolic syndrome(39) were observed. While neighborhood environment has been linked to lifestyle behaviors (e.g., dietary patterns, physical activity, and obesity), physical factors (e.g., pollution), and socio-environmental stressors (e.g., ethnic/racial discrimination) that precede chronic disease development, MASLD status may reflect distant biological effects of neighborhood environments. This indirect relationship could explain the study’s null findings concerning our perceived neighborhood variable. However, it is important to note that the majority of existing studies examine neighborhood influences on health risks use census-derived measures (e.g., socioeconomic deprivation) at the census tract of residence rather than subjective assessments of neighborhood environment.(27) Therefore, it could be possible that the documented mixed results of the effects of neighborhood environment on MASLD status might be explained by differences in the approach through which neighborhood environment is assessed.

Neighborhood environment, specifically perceived neighborhood violence, was negatively associated with ALoad. These findings are paradoxical as previous work indicates that neighborhood conditions can act as chronic stressors, resulting in higher levels of ALoad.(16, 40) According to the Transactional Stress Model (TSM), stress can be understood as the interaction between individuals and their environments and that the stress response is determined by an individuals’ appraisal process.(41) In order for perceived neighborhood violence to act as a stressor, it has to be appraised as a threat.(42) This is of note, as in the current study, farmworkers reported low levels of perceived neighborhood violence (*M* = *5*.4 ± 2.2). In fact, it has been previously shown that Southern Yuma County residents perceive their neighborhoods to be safe and calm relative to alternative locales residents have lived. which influenced their decision to move to or stay in the region.(43) Coping strategies may be associated with ALoad levels.(44) It has been found that while decreased use of active coping strategies contributes to higher levels of ALoad.(45), avoidance coping is associated with higher levels of stress which might negatively impact levels of ALoad.(46). Future research might investigate how the appraisal of and use of coping strategies to deal with neighborhood-level stressors, measured at individual and geospatial levels, influence Mexican-origin farmworkers’ ALoad levels.

The effect of perceived neighborhood violence on farmworkers’ MASLD status was negatively mediated by ALoad in our data. These findings are atypical, as most other data suggests a positive association between ALoad and increased risk for MASLD risk factors.(47, 48) Our data may reflect that protective social-cultural resources might dampen the negative impact of ALoad in Mexican-origin farmworkers’ risk for MASLD. According to the Stress Buffering Hypothesis, availability and perception of social support diminishes the negative health effects of stress by reducing exposure to stressors, attenuating the stress response, and bolstering the coping response.(49) This is of relevance, as farmworkers with confirmed MASLD reported high levels of perceived neighborhood social cohesion (*M* = 13.1 ± 2.7) and perceived neighborhood activities with neighbors (*M* = 12.4, ± 3.9). Notably, it has been previously described as a sense of collective efficacy among Southern Yuma County residents that may carry positive implications for health.(43) New research might seek to clarify pathways through which social-cultural resources might influence the relationship between ALoad and NAFLD risk among Mexican-origin farmworkers.

### Strengths and Limitations

There are several limitations of this study that warrant discussion. The primary limitation of the study was the cross-sectional nature of the data. As a consequence, our mediational analysis was guided by biologic plausibility, theory, and prior findings, rather than prospectively examining paths between neighborhood context, ALoad, and MASLD. Longitudinal examination of these factors would further strengthen causal interpretations among our models’ variables. Another limitation of this study is the fact that neighborhood context data was based on individuals’ self-report instead of neighborhood-level variables such as objective-built environment assessments. However, existent research suggests that subjective measures of neighborhood context have critical relationships with a range of health outcomes.(50) We suggest a combination of compositional and contextual factors representative of both the physical and social environments, and measured at multiple levels, to fully understand the impact of neighborhood context on farmworkers’ risk for MASLD. Also given data was collected from a focused geographic area along the U.S./Mexico border with a particularly high-density of farmworkers, and the mix of transitory and residentially stability of Yuma’s farmworker community, patterns observed here may not be like other farmworker communities. In addition, the project data was collected during the off-season so this sample may not be more representative of the more residentially stable Yuma County farmworker community. Despite these limitations, this study identified significant relationships among neighborhood context variables, ALoad and MASLD status in Mexican-origin farmworker.

## Conclusion

This study contributes to an emerging body of literature on the association of neighborhood environment and Mexican-origin farmworkers’ risk for MASLD. Paradoxically, it was found that perceived neighborhood violence was negatively associated with ALoad, though it may be these persons are physically active and engaged in their community, contributing to those perceptions. We also found ALoad was found to be a mediator between perceived neighborhood violence and MASLD status. Our findings may help inform policy efforts as well as the development of culturally appropriate strategies to reduce farmworkers’ risk for MASLD, which are highly responsive to the structural and systemic forces that shape the lived experience for Mexican-origin farmworkers. Future longitudinal and multi-level research is also important to investigate the pathways through which neighborhood environment and ALoad interact to affecting Mexican-origin farmworkers’ risk for MASLD.

## Data Availability

The data presented in this study are available on request from the corresponding author. The data are not publicly available due to the sensitive nature of the genetic information collected as part of the study.
